# Sleep Patterns and the Risk of Acute Stroke: Results From the INTERSTROKE International Case-Control Study

**DOI:** 10.1212/WNL.0000000000207249

**Published:** 2023-05-23

**Authors:** Christine Eileen Mc Carthy, Salim Yusuf, Conor Judge, Alberto Alvarez-Iglesias, Graeme J. Hankey, Shahram Oveisgharan, Albertino Damasceno, Helle Klingenberg Iversen, Annika Rosengren, Alvaro Avezum, Patricio Lopez-Jaramillo, Denis Xavier, Xingyu Wang, Sumathy Rangarajan, Martin O'Donnell, for INTERSTROKE

**Affiliations:** From the Department of Medicine (C.E.M.C., C.J., A.A.-I., M.O.D.), University of Galway, Ireland; Department of Medicine (S.Y., S.R.), Population Health Research Institute, McMaster University and Hamilton Health Sciences, Ontario, Canada; School of Medicine and Pharmacology (G.J.H.), Faculty of Health and Medical Sciences, University of Western Australia, Perth; Department of Medicine (S.O.), Rush Alzheimer Disease Research Center in Chicago, IL; Faculty of Medicine (A.D.), Eduardo Mondlane University, Maputo, Mozambique; Department of Neurology (H.K.I.), Rigshospitalet, University of Copenhagen, Denmark; Department of Molecular and Clinical Medicine (A.R.), University of Gothenburg and Region Västra Götaland, Sahlgrenska University Hospital, Gothenburg, Sweden; Department of Medicine (A.A.), International Research Center, Hospital Alemão Oswaldo Cruz, São Paulo, Brazil; Department of Medicine (P.L.-J.), Instituto de Investigaciones MASIRA, Universidad de Santander, Bucaramanga, Colombia; Department of Medicine (D.X.), St John's Medical College and Research Institute, Bangalore, India; and Department of Medicine (X.W.), Beijing Hypertension League Institute, China.

## Abstract

**Background and Objectives:**

Symptoms of sleep disturbance are common and may represent important modifiable risk factors of stroke. We evaluated the association between a spectrum of sleep disturbance symptoms and the risk of acute stroke in an international setting.

**Methods:**

The INTERSTROKE study is an international case-control study of patients presenting with first acute stroke and controls matched by age (±5 years) and sex. Sleep symptoms in the previous month were assessed through a questionnaire. Conditional logistic regression estimated the association between sleep disturbance symptoms and acute stroke, expressed as odds ratios (ORs) and 95% CIs. The primary model adjusted for age, occupation, marital status, and modified Rankin scale at baseline, with subsequent models adjusting for potential mediators (behavioral/disease risk factors).

**Results:**

Overall, 4,496 matched participants were included, with 1,799 of them having experienced an ischemic stroke and 439 an intracerebral hemorrhage. Short sleep (<5 hours: OR 3.15, 95% CI 2.09–4.76), long sleep (>9 hours: OR 2.67, 95% CI 1.89–3.78), impaired quality (OR 1.52, 95% CI 1.32–1.75), difficulty getting to sleep (OR 1.32, 95% CI 1.13–1.55) or maintaining sleep (OR 1.33, 95% CI 1.15–1.53), unplanned napping (OR 1.48, 95% CI 1.20–1.84), prolonged napping (>1 hour: OR 1.88, 95% CI 1.49–2.38), snoring (OR 1.91, 95% CI 1.62–2.24), snorting (OR 2.64, 95% CI 2.17–3.20), and breathing cessation (OR 2.87, 95% CI 2.28–3.60) were all significantly associated with an increased odds of acute stroke in the primary model. A derived obstructive sleep apnea score of 2–3 (2.67, 2.25–3.15) and cumulative sleep symptoms (>5: *5.38, 4.03–7.18*) were also associated with a significantly increased odds of acute stroke, with the latter showing a graded association. After an extensive adjustment, significance was maintained for most of the symptoms (not difficulty getting to/maintaining sleep and unplanned napping), with similar findings for stroke subtypes.

**Discussion:**

We found that sleep disturbance symptoms were common and associated with a graded increased risk of stroke. These symptoms may be a marker of increased individual risk or represent independent risk factors. Future clinical trials are warranted to determine the efficacy of sleep interventions in stroke prevention.


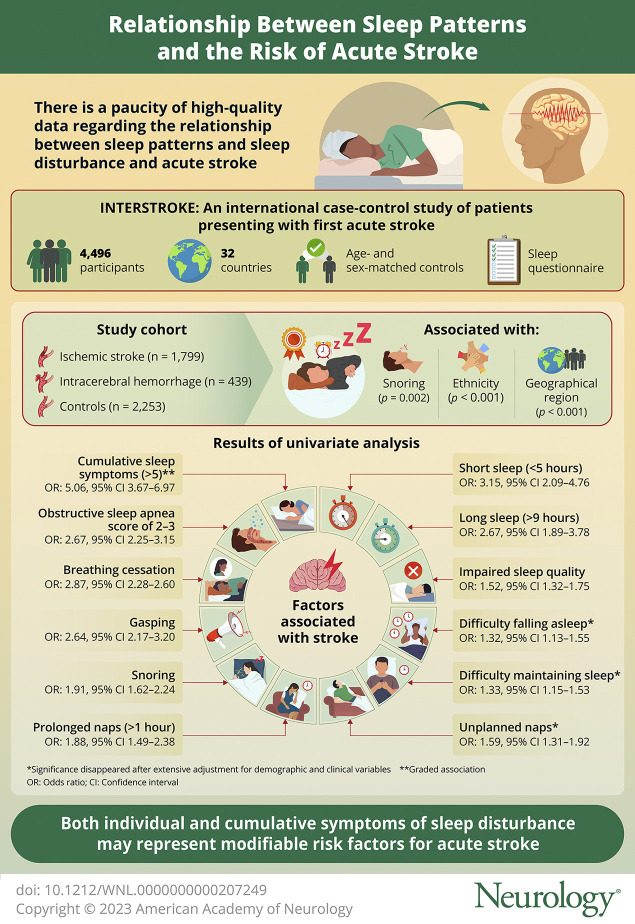


Adequate sleep is essential to health.^1^ Impairments in sleep represent a spectrum of disturbance, from mild deviations in duration to impairments in different domains (quality, initiation, and maintenance), associated symptoms (napping, snoring, snorting, and breathing cessation) through to complex syndromes. While there is convincing evidence of an association between obstructive sleep apnea (OSA) and stroke, the association of other sleep disorders or impairments in sleep are less certain.^2^ Given the high global prevalence of symptoms, the latter association, particularly, may represent an important modifiable target for population-level interventions in stroke prevention.

Prior epidemiologic studies have evaluated the association of these sleep parameters and stroke, but the methodology and results are inconsistent.^3-12^ Most studies have incompletely measured all relevant sleep domains, which precludes a thorough understanding of their independent contribution. Certain symptoms, such as nocturnal awakening and snorting, have also been interrogated infrequently, as potential independent risk factors.^12,13^ A further limitation is that most studies have been confined to single countries, with underrepresentation of populations from many regions of the world. In the INTERSTROKE study (a large international case-control study of risk factors of stroke), we evaluated the association of different symptoms of sleep impairment, individually and cumulatively, with the risk of first acute stroke.

## Methods

### Population

The INTERSTROKE study is a large international case-control study of patients presenting with first stroke and matched controls. The study design has been described in detail elsewhere.^14^ In brief, cases were required to meet the World Health Organization clinical definition for acute stroke, were within 72 hours of admission and 5 days of current symptom onset or “last seen without deficit,” and had a CT or an MRI available or planned within 1 week of presentation. Stroke was further defined as either ischemic stroke or intracranial hemorrhage (ICH), by hour of onset and whether symptoms occurred on waking (Supplemental Section 1, links.lww.com/WNL/C721). Patients with severe stroke or aphasia (unable to communicate effectively) were included if a valid proxy respondent was available. At least 1 control, without a stroke history, was recruited for each case, from either a community or hospital/outpatient setting. Controls were matched for sex and age (±5 years) in addition to ethnicity in countries where there was a substantial representation from multiple ethnic groups (e.g., South Africa, Canada). A standardized questionnaire was administered by trained research staff, which collected information about known and potential risk factors. Nonfasting blood samples were collected from participants and clinical measurements (e.g., blood pressure [BP], weight) were recorded during interview and/or from the participants notes. A supplementary questionnaire addressing sleep practices in the previous month was introduced in July 2012, with implementation in consecutive patients once adopted in centers. Matched cases/controls who undertook this questionnaire were the participants of the primary analysis in this study.

### Sleep Questionnaire

Specific questions asked are detailed in the eAppendix (eTable 1, links.lww.com/WNL/C721). In summary, participants were asked about sleep behaviors in the previous month (the month before stroke in cases). Areas addressed included the following: hours of nocturnal sleep duration (given in whole numbers, to the closest number of hours), sleep quality, sleep-onset latency (SOL), nocturnal awakening, sleeping during the day (both duration and whether it was planned), snoring, snorting or gasping, and breathing cessation or choking during sleep. Sleep duration categories were established for individual hour durations, truncated at <5 and >9. The reference level was set at 7 hours, based on univariate exploration of data and findings from previous research.^15,16^ The categorization of other individual sleep symptoms are detailed in the eAppendix (eTable 1). For symptoms of snoring, snorting, and breathing cessation during sleep, we derived an OSA score (range 0–3), with lower scores signifying a lower probability of sleep apnea (eTable 2). Post hoc, we also derived a summary count variable, based on the cumulative presence of sleep impairment symptoms. Termed Sleep Disturbance Symptom Burden, it included all sleep variables that were individually associated with stroke risk on univariate analysis (eTable 2). Sleep Disturbance Symptom Burden ranged from 0 to 9, with higher scores relating to greater numbers of sleep impairment symptoms.

### Covariates

Further covariates of interest were determined a priori in the statistical analysis plan. Both potential confounders and potential partial mediators were selected to be included in additive models. Occupation was categorized as “house wife,” “farmer,” “laborer,” “business,” “professional,” and “other.” Marital status was defined as either “currently married or living with partner” or “separated/not currently married.” Modified Rankin scale (mRS) score at baseline was categorized as “0,” “1,” or “>1.” Alcohol consumption was categorized as “never/former,” “low/moderate,” and “high intake/binge,” whereby a weekly intake of 1–14 drinks for women and 1–21 drinks for men was determined low/moderate, and >14 drinks for women or >21 drinks for men was determined high intake/binge.^17^ Leisure physical activity was categorized as “mainly active” and “mainly inactive,” with mainly active defined as moderate or strenuous leisure activity for ≥4 hours per week. Diet quality was defined by the modified Alternative Healthy Eating Index (AHEI).^18^ Waist-to-hip ratio (WHR) and body mass index (BMI) were obtained through anthropometric measurement. Depressive symptoms were determined based on the response to the question “During the last 12 months, was there ever a time when you felt sad, blue, or depressed for 2 weeks or more in a row?” Global stress was categorized as “none or some periods of stress” and “several periods or permanent stress.” Hypertension was present if the participant had a medical history of hypertension or adjusted BP of >140/90 mm Hg at admission. Diabetes was defined as a history of diabetes or a hemoglobin A1c ≥ 6.5%, and a history of both OSA diagnosis and atrial fibrillation/flutter were obtained.

### Statistical Analysis

Demographic characteristics were described by mean values and SDs or proportions, as appropriate. Distributions were analyzed through the Kruskal-Wallis rank sum test, Pearson χ^2^ test, and Fischer exact test, as appropriate.

We used univariate and multivariable conditional logistic regression analyses to determine the association of individual sleep domains with odds of acute stroke. For each sleep domain, we selected reference categories based on which category was associated with lowest odds of stroke on univariate analyses. Multivariable adjustment was conducted with additive models. Model 1 was univariate. Model 2 (primary model) was adjusted for age, marital status, occupation, and mRS at baseline. This was selected as the primary model because variables in subsequent models might include factors along the causal pathway. Model 3 was further adjusted for behavioral risk factors that might mediate risk (alcohol consumption, smoking history, leisure physical activity, AHEI score, WHR, BMI (kg/m^2^), depressive symptoms, and global stress) and model 4 further adjusted for disease risk factors that might mediate risk (hypertension, diabetes, a history of atrial fibrillation/flutter, and a diagnosis of OSA). Missing information of individual models was assessed, and missing data were not imputed. These models were also completed for the association of sleep duration, OSA score, and Sleep Disturbance Symptom Burden with ICH and ischemic stroke. Excluding controls, case-case analysis was also undertaken to determine whether these sleep domains had a stronger magnitude of association with ICH, compared with ischemic stroke.

We used the Wald likelihood ratio test to test for interactions between sleep duration and other sleep parameters and sleep duration and other predefined demographic variables (age, sex, region, and ethnicity). If a significant interaction was found, subgroup/stratified analyses were undertaken for large groups (≥1,000 available in unmatched cases/controls). Subgroup and case-case analysis were also undertaken for sleep domains (duration, OSA score, and Sleep Disturbance Symptom Burden) and hour of onset/wake-up variables. This was to observe whether the effect of sleep disturbance was influenced by the stroke onset's proximity to sleep.

Sensitivity analysis included subgroup analysis by source of control (i.e., hospital or community) and the exclusion of cases with proxy respondents. Post hoc, additional adjustment for household income was also undertaken. Statistical significance was defined as a 2-tailed *p* value of ≤0.05. All statistical analyses were undertaken using R statistical software, version 1.3.959.^19^

### Standard Protocol Approvals, Registrations, and Patient Consents

The INTERSTROKE study was approved by the ethics committees in all participating centers or countries, and participants (or proxy) provided written informed consent.

### Data Availability

Information on the design and rationale of INTERSTROKE has been previously published.^14^ Individual participant data, or other documents, will not be made available now.

## Results

### Demographic Characteristics

Overall, there were 4,496 matched participants, including 1,799 cases with ischemic stroke and 439 cases with ICH (16.7% of the INTERSTROKE population). The number that answered questions relating to sleep domains varied minimally (eTable 3, links.lww.com/WNL/C721). Distribution of demographic, risk factor, and sleep variables in matched cases and controls are described in Table 1.

**Table 1:**
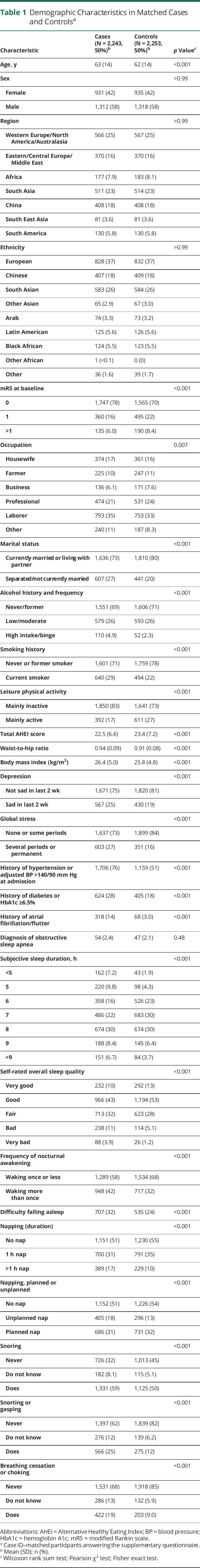
Demographic Characteristics in Matched Cases and Controls^a^

### Association of Sleep Duration With Acute Stroke

**Figure 1:**
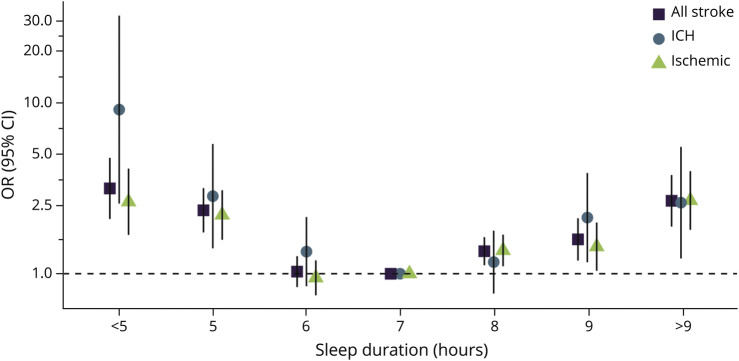
Odds of All Stroke, ICH, and Ischemic Stroke* *Conditional logistic regression adjusted for age, occupation, marital status, and mRS at baseline (primary model). OR presented on a log scale. ICH = intracranial hemorrhage; mRS = modified Rankin scale; OR = odds ratio.

In our primary multivariable model, short nocturnal sleep duration (<5 hours: odds ratio [OR] 3.15, 95% CI 2.09–4.76) and long nocturnal sleep duration (>9 hours: OR 2.67, 95% CI 1.89–3.78) were associated with an increased odds of all stroke, compared with 7 hours (reference). A significant U-shaped association persisted in all multivariable models, with similar associations found in relation to both ischemic stroke (<5 hours: OR 2.64, 95% CI 1.69–4.12; >9 hours: OR 2.68, 95% CI 1.81–3.98) and ICH (<5 hours: OR 9.12, 95% CI 2.57–32.34; >9 hours: OR 2.60, 95% CI 1.23–5.52) (Figure 1, Table 2). Odds of ICH and ischemic stroke did not differ in case-case analysis (*p* > 0.4; eTable 4, links.lww.com/WNL/C721).

**Table 2:**
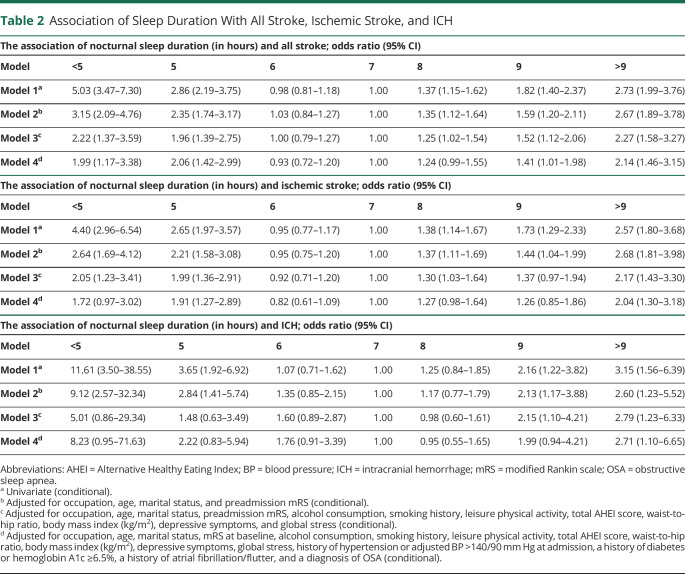
Association of Sleep Duration With All Stroke, Ischemic Stroke, and ICH

### Napping

**Figure 2:**
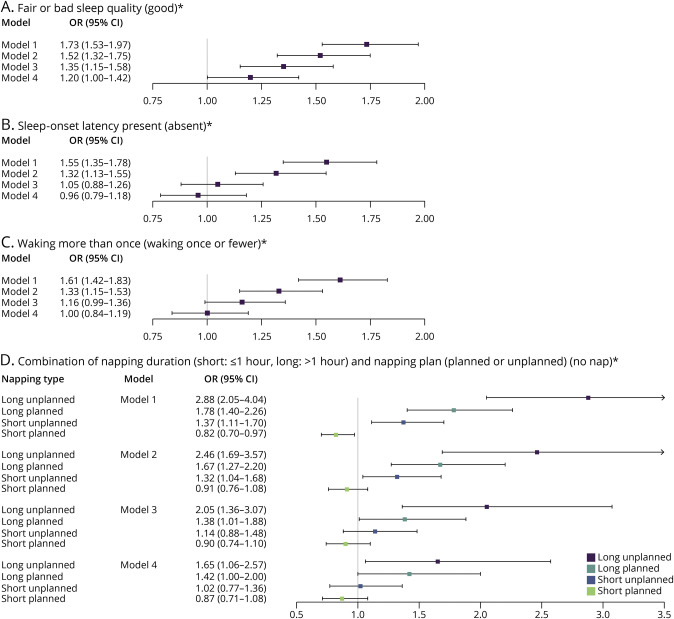
Odds of All Stroke for Poor or Fair Sleep Quality, Sleep-onset Latency, Waking More Than Once, and Napping Status Adjustment models are as follows: model 1: univariate (conditional); model 2: adjusted for occupation, age, marital status, and mRS at baseline (conditional); model 3: adjusted for occupation, age, marital status, mRS at baseline, alcohol consumption, smoking history, leisure physical activity, total AHEI score, waist-to-hip ratio, body mass index (kg/m^2^), depressive symptoms, and global stress (conditional); model 4: adjusted for occupation, age, marital status, mRS at baseline, alcohol consumption, smoking history, leisure physical activity, total AHEI score, waist-to-hip ratio, body mass index (kg/m^2^), depressive symptoms, global stress, a history of hypertension or adjusted BP >140/90 mm Hg at admission, a history of diabetes or hemoglobin A1c ≥6.5%, a history of atrial fibrillation/flutter, and a diagnosis of OSA (conditional). *For all panels (A–D), the reference level for each individual sleep parameter is displayed in brackets. AHEI = Alternative Healthy Eating Index; BP = blood pressure; mRS = modified Rankin scale; OR = odds ratio; OSA = obstructive sleep apnea.

Long duration (>1 hour) and unplanned napping were associated with significantly increased odds of all stroke in the primary model, while short duration (≤1 hour) and planned napping were not associated with an increased odds of all stroke. On further adjustment for potential mediators, napping of long duration remained significantly associated with stroke (eFigure 1, eFigure 2, links.lww.com/WNL/C721). A combined analysis of nap duration and planning are reported in Figure 2, with highest OR associated with long (>1 hour) unplanned napping (OR 2.46, 95% CI 1.69–3.57), and lowest OR associated with short (≤1 hour) planned napping (OR 0.91, 95% CI 0.76–1.08), in the primary model.

### Sleep Quality, SOL, and Nocturnal Awakening

In our primary model, self-reported poor or fair sleep quality (OR 1.52, 95% CI 1.32–1.75), SOL (OR 1.32, 95% CI 1.13–1.55), and frequent waking (OR 1.33, 95% CI 1.15–1.53) were associated with an increased odds of acute stroke. On further adjustment for potential mediators, sleep quality alone remained significantly associated with an increased odds of stroke (Figure 2).

### Symptoms of OSA

**Figure 3:**
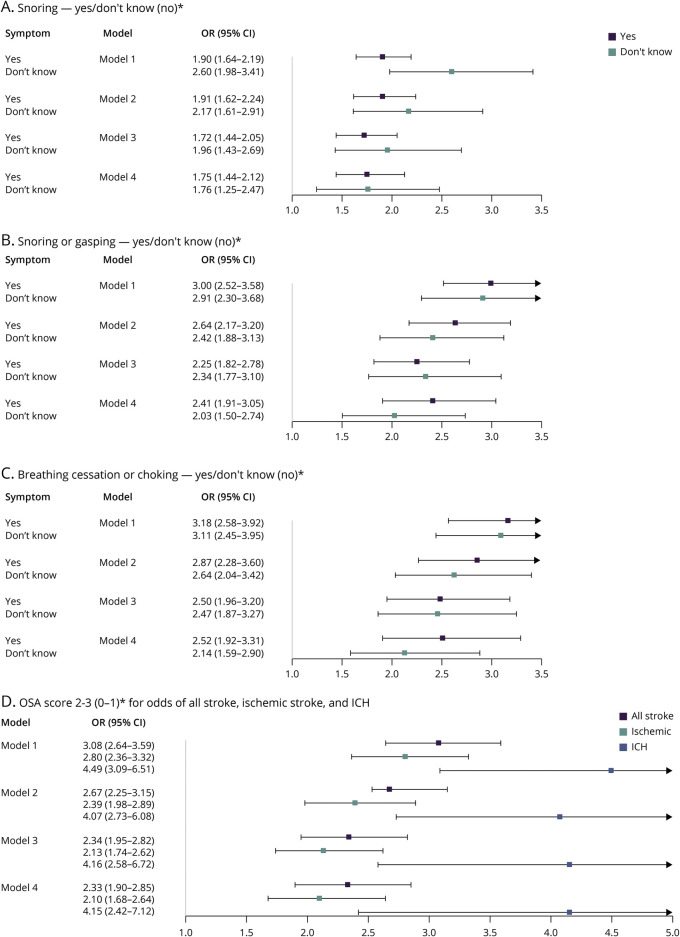
Odds of All Stroke for Snoring, Snorting, Breathing Cessation, and OSA Score Panels A–C demonstrate odds of all stroke for symptoms of, or uncertainty surrounding, snoring, snorting, or gasping and breathing cessation, respectively. Panel D demonstrates the odds of all stroke, ischemic stroke, and ICH with a potential OSA score of 2–3. Adjustment models are as follows: model 1: univariate (conditional); model 2: adjusted for occupation, age, marital status, and mRS at baseline (conditional); model 3: adjusted for occupation, age, marital status, mRS at baseline, alcohol consumption, smoking history, leisure physical activity, total AHEI score, waist-to-hip ratio, body mass index (kg/m^2^), depressive symptoms, and global stress (conditional); model 4: adjusted for occupation, age, marital status, mRS at baseline, alcohol consumption, smoking history, leisure physical activity, total AHEI score, waist-to-hip ratio, body mass index (kg/m^2^), depressive symptoms, global stress, a history of hypertension or adjusted BP >140/90 mm Hg at admission, a history of diabetes or hemoglobin A1c ≥6.5%, a history of atrial fibrillation/flutter, and a diagnosis of OSA (conditional). *For all panels (a-d), the reference level for each individual sleep parameter is displayed in brackets. AHEI = Alternative Healthy Eating Index; BP = blood pressure; ICH = intracerebral hemorrhage; mRS = modified Rankin scale; OR = odds ratio; OSA = obstructive sleep apnea.

Self-reported snoring (OR 1.91, 95% CI 1.62–2.24), snorting (OR 2.64, 95% CI 2.17–3.20), and breathing cessation (OR 2.87, 95% CI 2.28–3.60) were associated with statistically significant increased odds of all stroke in the primary model, maintaining significance with all further adjustment. We observed a similar magnitude of association for responding “do not know” as responding positively for these symptoms (Figure 3). An OSA score of 2–3 was associated with a significantly increased odds of all stroke (OR 2.67, 95% CI 2.25–3.15), ICH (OR 4.07, 95% CI 2.73–6.08), and ischemic stroke (OR 2.39, 95% CI 1.98–2.89), in the primary model, maintaining significance with subsequent adjustment. Odds of ICH vs ischemic stroke did not differ significantly in case-case analysis (*p* = 0.23).

### Sleep Disturbance Symptom Burden

**Figure 4:**
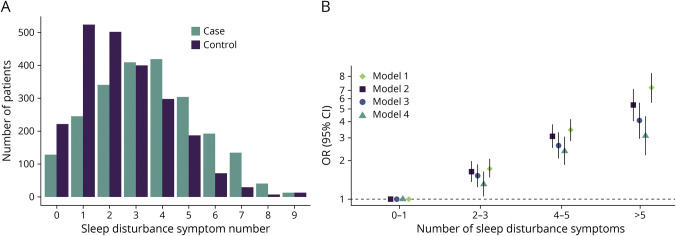
Sleep Disturbance Symptom Burden (A) Cumulative number of sleep disturbance symptoms in cases and controls, where sleep disturbance symptoms include the following: sleeping for <6 or >7 hours at night, SOL, waking more than once, napping for >1 hour, unplanned napping, and presence or uncertainty surrounding snorting, snorting or gasping, and breathing cessation or choking. (B) Odds of all stroke in sleep disturbance symptom number categories. OR presented on a log scale. Model 1 is univariate (conditional); model 2 adjusts for occupation, age, marital status, and mRS at baseline (conditional); model 3 adjusts for occupation, age, marital status, mRS at baseline, alcohol consumption, smoking history, leisure physical activity, total AHEI score, waist-to-hip ratio, body mass index (kg/m^2^), depressive symptoms, and global stress (conditional); model 4 adjusts for occupation, age, marital status, mRS at baseline, alcohol consumption, smoking history, leisure physical activity, total AHEI score, waist-to-hip ratio, body mass index (kg/m^2^), depressive symptoms, global stress, a history of hypertension or adjusted BP >140/90 mm Hg at admission, a history of diabetes or hemoglobin A1c ≥6.5%, a history of atrial fibrillation/flutter, and a diagnosis of OSA (conditional). AHEI = Alternative Healthy Eating Index; BP = blood pressure; mRS = modified Rankin scale; OR = odds ratio; OSA = obstructive sleep apnea; SOL = sleep-onset latency.

In the analysis of Sleep Disturbance Symptom Burden, an increasing number of symptoms were associated with a graded increase in stroke risk (2–3: OR 1.63, 95% CI 1.36–1.96; 4–5: OR 3.08, 95% CI 2.49–3.80; and >5: OR 5.38, 95% CI 4.03–7.18), with reference 0–1, in the primary model (Figure 4, eTable 5, links.lww.com/WNL/C721). Findings were also consistent for ischemic stroke (>5: OR 5.06, 95% CI 3.67–6.97) and ICH (>5: OR 8.36, 95% CI 4.05–17.26) (eTable 5, eFigure 3). Odds of ICH and ischemic stroke did not differ in case-case analysis (*p* = 0.73). Results for Sleep Disturbance Symptom Burden with OSA symptoms not included in score calculation are outlined in the eAppendix (eTable 6).

### Hour of Stroke Onset and Wake-up Stroke

When cases/matched controls were divided into hour-of-onset categories and wake-up status categories (eFigure 4, links.lww.com/WNL/C721), individual estimates were highest in the night-time subgroup and the wake-up subgroup for both short sleep duration and Sleep Disturbance Symptom Burden of >5 (eTables 7–10). Differences were less pronounced for long sleep duration, and estimates in the OSA score of 2–3 category were higher in the morning and wake-up subgroups (eTables 7 and 8, eTables 11 and 12). CIs crossed, however, and differences were not statistically significant in case-case analysis, other than for short sleep duration, which was associated with an increased odds of night time vs morning stroke (*p* = 0.04; eTable 13).

### Interaction of Sleep Symptoms

We observed a significant interaction between sleep duration and snoring (*p* = 0.002), where estimates in the primary model were highest in snorers with short sleep duration (OR 4.04, 95% CI 3.12–5.25) and lowest in nonsnorers with long sleep duration (OR 1.39, 95% CI 1.13–1.69) (eTable 14, links.lww.com/WNL/C721). There was no significant interaction found between sleep duration and other individual sleep symptoms (eTable 15).

### Interaction in Demographic Variables

An interaction was found between sleep duration and both ethnicity (*p* < 0.001) and region (*p* < 0.001). In the primary model, the association of short sleep duration with stroke was highest for South Asian ethnicity and South Asia and nonsignificant for Chinese ethnicity and China (eTable 16 and 17, eFigure 5, links.lww.com/WNL/C721). The association between long sleep duration and stroke remained significant in all ethnicity and region subgroups examined (eTable 16 and 17, eFigure 5). There was no significant interaction between sleep duration and age or sex (eTable 15).

### Sensitivity Analysis

The source of information for cases (i.e., patient or proxy) did not materially alter findings for the sleep domains (eTable 18, links.lww.com/WNL/C721). Magnitude of ORs were generally higher for community vs hospital controls, but directions of estimates were consistent by source of control, in the primary model (eTables 19 and 20). Additional adjustment for household income did not materially change results (eTable 21).

## Discussion

In this large international case-control study, we found a significant association between sleep impairments and risk of acute stroke. The odds of acute stroke were increased with short and long sleep duration, poor sleep quality, symptoms of OSA (snoring, snorting, and breathing cessation) and prolonged napping, after an extensive adjustment. The magnitude of association was additive, with a graded increase in odds of acute stroke, for cumulative increase in sleep symptoms, with consistent findings for ischemic stroke and ICH. Impairments in sleep domains associated with an increased risk of stroke were common and may represent important modifiable targets for stroke prevention interventions.

Associations between acute stroke and both short and long sleep duration have been reported in previous observational research.^20^ In our study, there was a higher magnitude of association for short sleep duration, compared with long duration, on univariate analysis. However, after adjustment for coexisting risk factors, there was more marked attrition of the OR for short sleep duration. This suggests that confounders and mediators of these associations differ and that long sleep duration is more likely to have an independent association with acute stroke. In addition, the association for long sleep duration was found more consistently in different ethnicities and regions. This is complimentary to previous research, where associations have been more consistent for long sleep duration.^9-11^ The mechanisms underlying this association could be influenced by BP surges associated with sleep architecture changes, for example, or relate to lack of physiologic challenge.^21-23^ The relationship, however, may also be subject to residual universal confounding because long sleep duration may represent an epiphenomenon of comorbidity, older age, an otherwise sedentary lifestyle, and sedative use.^24-30^ A large proportion of participants in our study reported a nocturnal sleep duration within the range associated with stroke (<6 or >7 hours), making it an important potential risk factor for developing and evaluating public interventional studies.

Daytime sleeping (napping) has also been associated with an increased cardiovascular disease risk, particularly when prolonged, a finding that has challenged whether the common practice of siesta is healthy, with some conflicting results.^12,24,27,30-33^ The Prospective Urban Rural Epidemiology study found that long duration napping was significantly associated with an increased risk of major cardiovascular events, in those who slept longer than 6 hours at night.^34^ While, by contrast, we found no significant interaction with sleep duration, our findings, in another international setting, also suggest that the association with daytime napping is contextual. Long duration and unplanned napping are potential risk factors of stroke, but short duration and planned napping (e.g., siesta) were not associated with an increased risk, with the point estimate directionally suggestive of reduced odds. While our findings merit reproduction, they may help inform patient advice and management, where a prolonged nap may be harmful or representative of an underlying condition that requires further work-up, and a brief planned nap is less likely to increase the risk of stroke.

OSA is an established risk factor of acute ischemic stroke.^35^ Our findings suggest that individual symptoms, which may represent OSA, are independently associated with stroke. This is complimentary to previous research, where snoring has been studied more frequently than snorting and breathing cessation.^4,8,12,36^ Patients with these self-reported symptoms of OSA may be at risk of stroke, independent of OSA severity and treatment, as has been suggested by a recent post hoc analysis of the SAVE trial.^36^ Symptoms were common before stroke, in our study, where 59% of cases reported snoring, with 25% and 19% reporting snorting and breathing cessation, respectively. In addition, participants who were unsure about OSA-related symptoms had a similar increased odds of stroke as participants who endorsed these symptoms, suggesting a potential lack of awareness of OSA symptoms, rather than absence. This has important implications for questionnaire-based OSA screening in clinical practice and for conducting and interpreting clinical research in this context. While randomized controlled trials looking at positive airway pressure (PAP) in OSA have not reproduced evidence of lower cardiovascular events found in observational studies, there does seem to be a potential risk reduction when adherence is high and when PAP is used >4 hours per night.^37-39^ Given the frequency of symptoms in our study, and their relationship with stroke, exploring interventions that improve PAP adherence may be of benefit in primary stroke prevention. Identifying subgroups that are more likely to benefit, in addition to exploring alternative management strategies in patients with self-reported symptoms, are also important areas for future research.

Sleep quality, a self-reported subjective measure, was fair or poor in 46% of patients with acute stroke, in the preceding month. Further individual symptoms of frequent nocturnal awakening and SOL were also common, self-reported in 42% and 32% of cases, respectively. In our primary model, each of these sleep impairments was associated with an increased odds of acute stroke. However, after full adjustment, the latter 2 exposures were no longer significantly associated, similar to that observed in previous research.^5,13,24,26,40,41^ These results imply that the association was confounded or mediated by cerebrovascular risk factors. The challenge in interpretation is to determine which of these adjustment variables are confounders and which reside along a potential causal pathway. For example, increased alcohol intake may cause disruptions in sleep quality, but impairments in sleep quality may result in increased use of alcohol, as a sedative.^42^ Our primary model, which did not include cerebrovascular risk factors, is expected to overestimate the independent association of sleep impairment and stroke risk, while the fully adjusted model likely underestimates the association, given that mediator variables are included. While numerous observational studies have reported bidirectional associations between sleep impairment and cerebrovascular risk factors, interventional clinical trials are required to determine whether improvement in sleep quality results in prospective changes in these factors and subsequent disease risk.^43^ Few trials have been conducted to determine the effect of behavioral sleep interventions on cerebrovascular risk factors to date, with overall results being inconclusive.^44^ The SLEPT trial, a phase IIb trial reported modest improvements in sleep quality with digital cognitive behavioral therapy (dCBT-I) but did not effect change in BP.^45^ The results of the recent DISCO trial, however, where dCBT-I was associated with improvement in cognitive complaints, shows potential promise for this type of intervention in the cerebrovascular domain.^46^

The graded association between the accumulation of these outlined sleep disturbance symptoms and stroke also has important implications from a public health and interventional trial perspective, particularly given the magnitude of estimates. This is an extensive study of sleep disturbance in this context, confirming previous findings in a limited amount of previous studies.^4,5,41^ Even if the relationship is not causal, those with multiple sleep disturbance symptoms should be thought of as at high risk of stroke and important targets for primary prevention studies involving both traditional risk factor management and interventions for sleep disturbance.

Our study has a few potential limitations. First, sleep symptoms were ascertained through subjective reporting, and validated tools/scores were not used to examine sleep quality/OSA symptoms and their association with stroke. Our assessments of sleep practices were similar to those of previous research in this area, however, and undertaking a prolonged sleep questionnaire would not have been possible in the context of this multifaceted study.^24^ Second, because incident stroke may result in sleep disturbance, there is a risk of misclassification and recall bias.^47^ Uncertainty surrounding OSA symptoms may be subject to similar biases, with stroke patients potentially more likely to answer “do not know.” Efforts taken to reduce the risk of these biases included recruiting cases within 72 hours of hospital admission and asking about sleep over a short time frame. Prospective cohort studies in this area have generally asked about sleep practices over a longer time frame. Because sleep can change over time, our shorter time frame may have resulted in more accurate sleep symptom reporting.^24,48^ In addition, our results may be partially due to trigger effects of sleep disturbance on stroke, given the shorter time frame. Findings in relation to risk of night time and wake-up stroke may also be suggestive of this. Third, there may be a risk of healthy volunteer bias among community controls. In an effort to combat this, however, some hospital/outpatient controls were recruited. Given the findings of our sensitivity analysis, this inclusion likely biased our findings toward the null, underestimating the odds of stroke associated with sleep symptoms in a healthy community setting, with results more likely reflective of the true global population risk. Fourth, subgroup analyses were limited by participant numbers, and associations between sleep duration and stroke in different regions/ethnicities, for example, could not be fully assessed. Finally, despite extensive adjustment, residual cofounding cannot be excluded. We were also unable to adjust for shift work or sedative use, and levels of diagnosed OSA were low.

In conclusion, our results suggest that individual and cumulative symptoms of sleep disturbance may be important modifiable risk factors of stroke and/or their presence identifies individuals at an increased risk of stroke. Our findings also suggest a complex relationship of sleep impairment, intermediate cerebrovascular risk factors, and stroke risk. Given that individual sleep disturbance symptoms were common and associated with an increased odds of stroke, interventional studies in patients with high sleep disturbance burden, and in those with individual sleep symptoms, should be considered a priority research target in the global effort to reduce stroke incidence.

## Glossary

AHEI: Alternative Healthy Eating Index
BMI: body mass index
BP: blood pressure
dCBT-I: digital cognitive behavioral therapy
ICH: intracranial hemorrhage
mRS: modified Rankin scale
OR: odds ratio
OSA: obstructive sleep apnea
PAP: positive airway pressure
SOL: sleep-onset latency
WHR: waist-to-hip ratio
